# 

**Published:** 1863-10

**Authors:** 


### CONTENTS.

### ORIGINAL CONTRIBUTIONS.

ART. XXV.—The Remote Cause of Scrofulous Forms of Disease. By W. Byrd Powell, M.D., of Covington, Ky.,........................ 481

ART. XXVI.—Erysipelas, Diphtheria, and Scarlatina. By A. Fisher, M.D., of Chicago, Ill.,........................ 491

ART. XXVII.—Singular Case of Erysipelas. By W. H. Byford, A.M., M.D., Professor, &c., Chicago Medical College,........................ 495

ART. XXVIII.—Dentition, and its Connection with the Bowel-Affections of Children. By N. S. Davis, M.D., Chicago, Ill.,... 498

ART. XXIX.—Experiments with the Sulphite of Lime as an Antiseptic. By E. Andrews, M.D.. Professor of Surgery in Chicago Medical College,........................ 504

### SELECTIONS.

Lecture on Eczema, Including its Impetiginous, Lichenous, and Pruriginous Varieties. By T. McCall Anderson, M.D........................ 501

Lecture on Certain Diseases of the Brain and Nervous System, with special reference to the changes in opinion and practice which result from recent researches in physiology and pathology. By Charles Bland Radcliff, M.D.,........................ 516

### EDITORIAL.

A Treatise on Hygiene: with special reference to the Military Service. By William A. Hammond, Surgeon-General of U. S. Army; Fellow of the College of Physicians of Philadelphia, &c.,........................ 530

